# Evaluating the antidiabetic effects of Chinese herbal medicine: Xiao-Ke-An in 3T3-L1 cells and KKAy mice using both conventional and holistic omics approaches

**DOI:** 10.1186/s12906-015-0785-2

**Published:** 2015-08-13

**Authors:** Zhenzhong Yang, Linli Wang, Feng Zhang, Zheng Li

**Affiliations:** Pharmaceutical Informatics Institute, College of Pharmaceutical Sciences, Zhejiang University, Hangzhou, 310058 China; State Key Laboratory of Modern Chinese Medicine, Tianjin University of Traditional Chinese Medicine, Tianjin, 300193 China

**Keywords:** Type 2 diabetes mellitus, KKAy mice, 3T3-L1 cells, Xiao-Ke-An, TCM, Network pharmacology

## Abstract

**Background:**

Xiao-Ke-An (XKA) is a Chinese medicine widely used for treating type 2 diabetes mellitus (T2D). It is composed of eight herbal medicines traditionally used for T2D, including *Rehmannia glutinosa* Libosch, *Anemarrhena asphodeloides* Bunge, *Coptis chinensis* Franch, etc. The aim of the present study was to investigate the antidiabetic effects of XKA with both conventional and holistic omics approaches.

**Methods:**

The antidiabetic effect of XKA was first investigated in 3T3-L1 cells to study the effect of XKA on adipogenesis *in vitro*. Oil Red O staining was performed to determine the lipid accumulation. The intracellular total cholesterol (TC) and triglyceride (TG) contents in XKA treated 3T3-L1 cells were also evaluated. The therapeutic effects of XKA was further evaluated in KKAy mice with both conventional and holistic omics approaches. Body weight, fasting and non-fasting blood glucose, and oral glucose tolerance were measured during the experiment. At the time of sacrifice, serum was collected for the measurement of TG, TC, high-density lipoprotein cholesterol (HDL-c) and low-density lipoprotein cholesterol (LDL-c). The liver, kidney, spleen, pancreas, heart and adipose tissues were harvested and weighted. The liver was used for further microarray experiment. Omics approaches were adopted to evaluate the holistic rebalancing effect of XKA at molecular network level.

**Results:**

XKA significantly inhibited adipogenic differentiation, lowered the intracellular TC and TG contents in 3T3-L1 cells. XKA improved the glucose homeostasis and lipid metabolism, ameliorated insulin resistance in KKAy mice. Futhermore, XKA also exhibited effective therapeutic effects by reversing the molecualr T2D disease network from an unbalanced state.

**Conclusions:**

This study investigated the antidiabetic effects of XKA with both conventional and holistic omics approaches, providing both phenotypic evidence and underlying action mechanisms for the clinical use of XKA treating T2D.

**Electronic supplementary material:**

The online version of this article (doi:10.1186/s12906-015-0785-2) contains supplementary material, which is available to authorized users.

## Background

Type 2 diabetes mellitus (T2D) is one of the fastest growing public health problems throughout the world affecting more than 366 million individuals globally [1]. Various antidiabetic medications are clinically available, including biguanides, thiazolidinediones, sulfonylureas, alpha-glucosidase inhibitors, incretin mimetics and enhancers, insulin [2]. These drugs treat T2D through different mechanisms such as reducing intestinal glucose absorption, lowering hepatic glucose output, enhancing pancreatic insulin secretion, improving insulin sensitivity and reversing peripheral glucose utilization [2]. Considering the complex mechanisms and pathophysiological abnormalities in T2D, it is extremely hard to reverse T2D disease state with monotherapies [3–5]. Thus combination therapy is becoming a promising alternative choice in clinical practice by designing drug combinations or compound drugs to interact with multiple targets and achieve synergistic treatment effects [6]. For example, traditional Chinese medicines (TCM) are attracting more attentions for their efficacy and less frequent side effects for treating T2D [7–10].

A number of antidiabetic efficacy indexes have been developed for T2D therapy, including body mass index (BMI), plasma glucose, total cholesterol (TC), triglyceride (TG), high-density lipoprotein cholesterol (HDL-c), insulin, glycated haemoglobin (HbA1c), etc [11]. However, most of these indexes only reflect a single aspect of the antidiabetic effects and could not reflect the comprehensive impacts caused by the compound drugs. A combination of markers might perform better than a single one [12]. Network pharmacology offers a molecular network view to understand the relationship between drugs and targets, providing a global perspective to investigate the drug efficacy [13]. Integrating network pharmacology analysis and ‘omics’ data enables the evaluation of the drug efficacy at the systems network level.

**Fig. 1:**
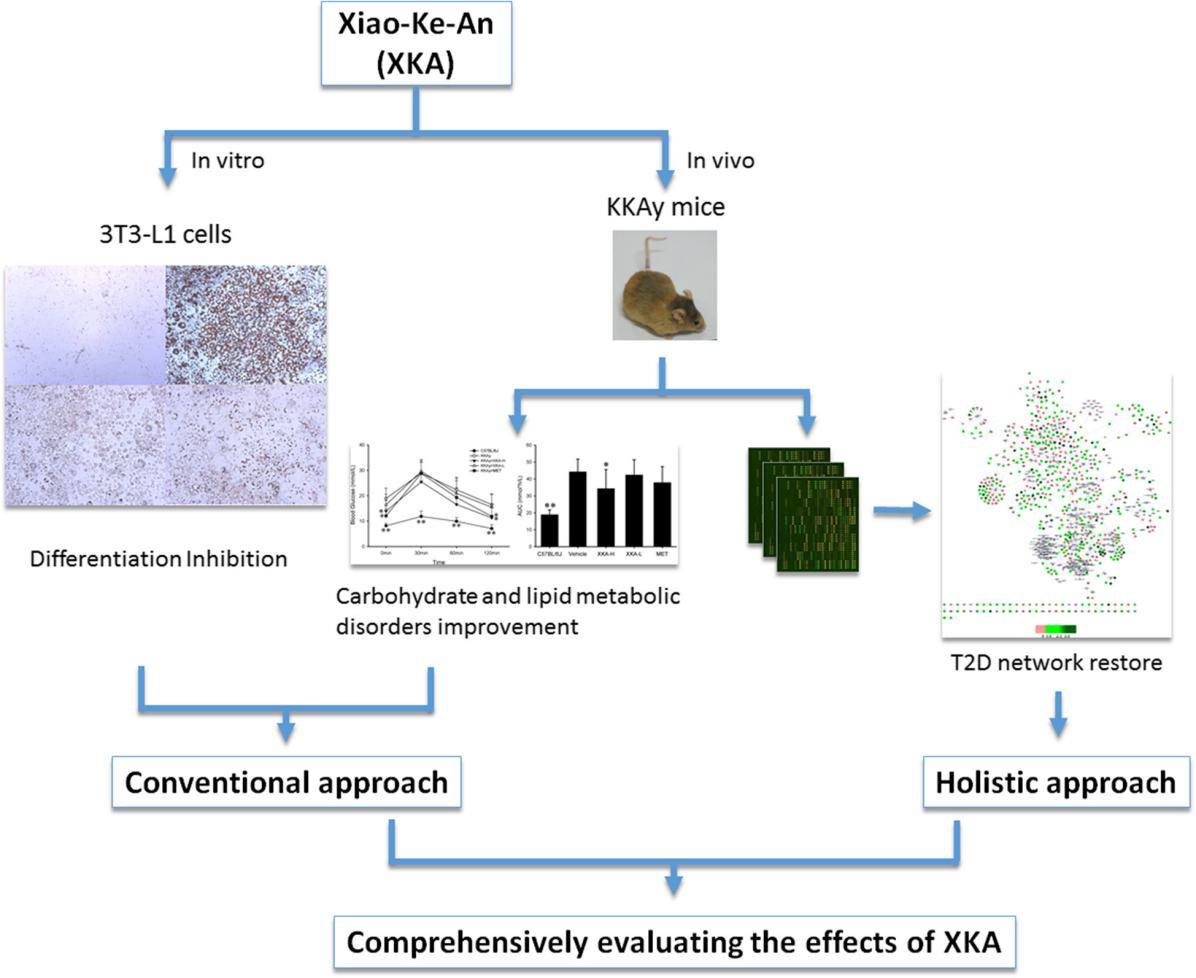
Framework of evaluating the antidiabetic effects of XKA with both conventional and holistic omics approaches

Xiao-Ke-An (XKA) is a traditional Chinese medicine for ‘Xiaoke’ symptom (the description of T2D in TCM), which was approved by the China Food and Drug Administration. In previous studies, XKA has been reported to alleviate hyperglycemia and hyperlipemia [14]. A more comprehensive evaluation of the pharmacological effects of XKA in T2D is needed for better understanding of its therapeutic effectiveness and action mechanisms. The 3T3-L1 cell is a well characterized cell line for investigating adipocyte differentiation and lipid accumulation, and KKAy mice is an excellent animal model of T2D, characterized by obesity, hyperglycaemia, dyslipidaemia, and insulin resistance. In this study, XKA was investigated for its therapeutic effects in 3T3-L1 cells and KKAy mice with both conventional and holistic omics approaches (Fig. 1).

## Methods

### Materials and chemicals

XKA includes *Rehmanniae radix*, *Anemarrhenae rhizome*, *Coptidis rhizome*, *Lycii cortex*, *Lycii fructus*, *Polygonati odorati rhizome*, *Ginseng radix et rhizoma* and *Salviae miltiorrhizae radix et rhizome,* with a 6:5:2:4:2:3:2:3 weight ratio of the eight herbal compositions. XKA was manufactured by Jilin Tonghua Huaxia Pharmaceutical Co. Ltd. (Jilin, China). Briefly, the *Ginseng radix et rhizome* and half of the *Anemarrhenae rhizome* were crushed into powder. *Coptidis rhizome* was extracted with water. The remaining half of the *Anemarrhenae rhizome* and the five other herbs were extracted with water together. Both extracts were concentrated together to syrup. Finally the powder was mixed to the syrup and dried.

Metformin was purchased from Melone Biotechnology Co. Ltd. (Dalian, China). Dexamethasone, 3-isobutyl-1-methylxanthine, dimethyl sulfoxide (DMSO), insulin, Oil Red O and 3-(4,5-dimethylthiazol-2-yl)-2,5-diphenyltetrazolium bromide (MTT) were purchased from Sigma-Aldrich Co. (USA). Dulbecco's Modified Eagle Medium (DMEM), bovine serum, fetal bovine serum (FBS), penicillin and streptomycin were purchased from Gibco (USA). Triglycerides kit and total cholesterol kit were purchased from BHKT Clinical Reagent Co., Ltd. (Beijing, China). BCA protein assay kit was purchased from Beyotime Institute of Biotechnology (Jiangsu, China).

### Preparation of XKA extracts

XKA was extracted twice with 70 % ethanol/H_2_O (1:10, w/v) for 1 h each time, and then concentrated in vacuum to obtain the extracts of XKA. The extracts of XKA were dissolved in DMSO with the final DMSO concentration less than 0.1 % for this *in vitro* experiments, and the same vehicle was used in the control group.

### 3T3-L1 cell culture

3T3-L1 preadipocytes were obtained from the Cell Bank of Type Culture Collection of the Chinese Academy of Sciences (Shanghai, China) and cultured in DMEM containing 10 % bovine serum and penicillin/streptomycin in a humidified atmosphere of 5 % CO_2_ at 37 °C.

### Cell viability assay

Cell viability was determined by MTT assay. The 3T3-L1 preadipocytes were seeded at 3 × 10^3^ cells/well in 96-well plates and incubated at 37 °C. After 2-day incubation, the cells were treated with different concentrations of XKA extracts or vehicle for 48 h. At the end, the medium was removed, and 100 μL of MTT solution (0.5 mg/mL) was added to the 3T3-L1 cells. After the wells were incubated for another 4 h at 37 °C, the medium was removed, and the synthesized formazan crystals were dissolved in 100 μL of DMSO. Finally, absorbance was measured at 580 nm using an infinite F200 microplate reader instrument (Tecan Group Ltd., Switzerland) (*n* = 3). Data was calculated as a percentage of MTT compared to control cells.

### 3T3-L1 cell differentiation and Oil Red O staining

The 3T3-L1 preadipocytes were induced to differentiate into adipocytes as described previously [15]. Briefly, the 3T3-L1 preadipocytes were seeded at 1 × 10^4^ cells/well in 48-well plates and proliferated to confluence. Adipogenesis was triggered with differentiation induction medium containing 0.5 mM 3-isobutyl-1-methylxanthine, 1.0 μM dexamethasone and 10 μg/mL insulin in DMEM with 10 % FBS. Three days later, cells were maintained in a differentiation maintenance medium containing 10 μg/mL insulin in DMEM with 10 % FBS for another 3 days. Next, the medium was replaced with DMEM containing 10 % FBS every 3 days. After 9–11 days’ incubation, more than 80 % cells were differentiated into adipocytes. Cells were treated with different concentrations of XKA extracts throughout the entire differentiation process. The 3T3-L1 adipocytes were fixed with 0.4 % paraformaldehyde (PFA) for 1 h. Then the cells were stained with Oil Red O solution for 0.5 h. The cells were washed with 70 % ethanol/H_2_O (v/v) twice. Appearance was recorded by an inverted fluorescence microscope Leica DMI 6000 B (Leica Microsystems, Wetzlar, Germany). Then, Oil Red O dye in lipid droplets was eluted into isopropanol. Finally, absorbance was measured at 492 nm using an infinite F200 microplate reader instrument (Tecan Group Ltd., Switzerland) (*n* = 3).

### TG and TC assay in 3T3-L1 cells

The process of differentiating 3T3-L1 preadipocytes into adipocytes were similar as mentioned previously. Briefly, the 3T3-L1 preadipocytes were seeded at 2 × 10^4^ cells/well in 24-well plates and proliferated to confluence, and the differentiation medium was replaced every 2 days. When more than 80 % cells were differentiated into adipocytes, cells were washed with phosphate buffer. The amount of intracellular triglyceride and total cholesterol was determined with the triglycerides kit and total cholesterol kit after cell fractured, respectively. TG and TC values were corrected by their protein content.

### Animals and treatment

Eight-week old male KKAy mice were obtained from Beijing HFK Bioscience Co., Ltd. (Beijing, China). Age and sex-matched non-diabetic C57BL/6J mice were obtained from Shanghai SLAC laboratory Animal Co., Ltd. (Shanghai, China).

Mice treated with XKA were based on our previous study [16], and more measurements and analysis were presented in this study. Briefly, all mice were housed in an environmentally controlled room at 22 ± 2 °C with a relative humidity of 55 ± 15 %, under a 12 h light/dark cycle. The KKAy mice were fed a high-fat diet as models of T2D [17], whereas C57BL/6J mice were fed normal chow diet as non-diabetic controls. All animals were allowed *ad libitum* access to solid food and water. They were used in experiments after 2 weeks of acclimation. All experimental procedures were approved by the Animal Ethic Review Committees of Zhejiang University.

Ten-week old KKAy mice were randomly divided into 4 groups according to fasting blood glucose values. Then, the KKAy mice were administered by oral gavage once daily with distilled water, XKA (1.5 g/kg), XKA (0.75 g/kg), or metformin (250 mg/kg), for a duration of 32 days. At the same time, the non-diabetic controls received distilled water.

During the experiment, body weight was monitored regularly. Blood glucose values were determined regularly for the non-fasting and fasting (after a 5 h fast) mice using the One-Touch Ultra blood glucose meter (Lifescan, CA, USA) by collecting blood from the tip of the tail vein. After 32 days of drug administration, blood samples were taken and mice were immediately sacrificed by cervical dislocation in the fed state. The liver, kidney, spleen, heart and adipose tissues were collected and weighted. The livers from non-diabetic controls, diabetic models and XKA (1.5 g/kg) treatment groups were stored in liquid nitrogen for further microarray experiment.

### Oral glucose tolerance test

After 30 days of treatment, an oral glucose tolerance test (OGTT) was performed. Briefly, after animals fasted for 5 h, glucose at a dose of 2.0 g/kg was then orally administered to the mice. The blood glucose levels were measured before and 30, 60, 120 min after glucose administration using a One-Touch Ultra blood glucose meter. The results of the OGTT were also expressed as area under the glycemia-versus-time curve (AUC). The AUC during the OGTT was calculated via the following equation:

1 $$ \mathrm{A}\mathrm{U}\mathrm{C}\ \left(\mathrm{mmol}*\mathrm{h}/\mathrm{L}\right) = 0.25 \times {\mathrm{BG}}_{0\  \min } + 0.5 \times {\mathrm{BG}}_{30\  \min } + 0.75 \times {\mathrm{BG}}_{60\  \min } + 0.5 \times {\mathrm{BG}}_{120\  \min }. $$

### Blood sample analysis

After 32 days of treatment, blood samples were obtained in the fasting state. Serum samples were separated from the blood and stored at -20 °C until measurement. Serum TG, TC, HDL-c and low-density lipoprotein cholesterol (LDL-c) measurements were carried out with a Hitachi 7600 Automatic Analyzer using appropriate kits. The atherogenic index [18] was calculated using the following equation:

2 $$ \mathrm{Atherogenic}\ \mathrm{index} = \left(\mathrm{T}\mathrm{C}\hbox{-} \mathrm{H}\mathrm{D}\mathrm{L}\hbox{-} \mathrm{c}\right)/\mathrm{H}\mathrm{D}\mathrm{L}\hbox{-} \mathrm{c} $$

### RNA extraction purification and quality assessment

The RNA extraction and quality evaluations were carried out as described before [16].

### Microarray experiment

Affymetrix mouse 430 2.0 chips were used. Whole genome microarray analysis was performed using Affymetrix products as described in our previous study [16]. Five mice per group were used for the microarray experiments. The dataset was deposited as CEL files to GEO and the access number is GSE62087.

### Microarray data analysis

Raw data were analyzed in ArrayTrack 3.5.0 [19], a java-based microarray analysis tool developed by the US FDA. Global scaling normalization was performed with Median Scaling Normalization using a target median value of 1000. Reverse rate (RR) [15] was used to evaluate the effect of XKA in reversing the changes of gene expression in diabetic models. It was calculated via the following equation:

3 $$ \mathrm{R}\mathrm{R} = \left({\mathrm{M}}_{\mathrm{i}}\hbox{-} {\mathrm{T}}_{\mathrm{i}}\right)/\left({\mathrm{M}}_{\mathrm{i}}\hbox{-} {\mathrm{N}}_{\mathrm{i}}\right) $$

In equation (3), N_i_, M_i_ and T_i_ refer to the average expressions of gene i in normal group, model group and XKA treatment group, respectively.

The network recovery index (NRI) [20] was used to quantitatively evaluate the network rebalance effect of drug treatment in the unbalanced molecular network of T2D. In our previous work, a T2D molecular network [21] was constructed, which was used for the NRI evaluation in this study. The process of NRI calculation was described previously [20]. Briefly, the regulating score (RS) and the ratio of recovery regulation (Rr) should be calculated first, and then NRI could be obtained.

Firstly, RS was calculated to evaluate the influence of T2D and XKA on each node in the network with equation (4).

4 $$ \mathrm{R}\mathrm{S}=\left\{\begin{array}{c}\hfill \frac{{\mathrm{E}}_{\mathrm{m}}\hbox{-} {\mathrm{E}}_{\mathrm{n}}}{{\mathrm{E}}_{\mathrm{n}}},\ \mathrm{f}\mathrm{o}\mathrm{r}\ \mathrm{diabetic}\ \mathrm{model}\hfill \\ {}\hfill \frac{{\mathrm{E}}_{\mathrm{t}}\hbox{-} {\mathrm{E}}_{\mathrm{n}}}{{\mathrm{E}}_{\mathrm{n}}},\ \mathrm{f}\mathrm{o}\mathrm{r}\ \mathrm{treatment}\kern2em \hfill \end{array}\right. $$

In equation (4), E_m_, E_t_ and E_n_ refer to the expression value of the diabetic model group, the drug treatment group and the normal group, respectively. In this study, nodes with RS (diabetic model) > 0.5 were considered as upregulated nodes, while RS (diabetic model) <−0.33 were defined as downregulated ones.

Rr was calculated as the ratio of nodes with recovery regulation by equation (5).

5 $$ \mathrm{R}\mathrm{r}={\displaystyle {\sum}_{\mathrm{i}\in \mathrm{M}}\frac{{\mathrm{R}}_{\mathrm{i}}}{\mathrm{M}}},\ \mathrm{where}\ {\mathrm{R}}_{\mathrm{i}}=\left\{\begin{array}{c}\hfill 1,\ \frac{{\mathrm{R}\mathrm{S}}_{\mathrm{i},\mathrm{m}}\hbox{-} {\mathrm{R}\mathrm{S}}_{\mathrm{i},\mathrm{t}}}{{\mathrm{R}\mathrm{S}}_{\mathrm{i},\mathrm{m}}}>0\hfill \\ {}\hfill 0,\ \frac{{\mathrm{R}\mathrm{S}}_{\mathrm{i},\mathrm{m}}\hbox{-} {\mathrm{R}\mathrm{S}}_{\mathrm{i},\mathrm{t}}}{{\mathrm{R}\mathrm{S}}_{\mathrm{i},\mathrm{m}}}\le \kern0.3em 0\hfill \end{array}\right. $$

In equation (5), RS_i,m_, RS_i,t_ refer to the RS value of node i in diabetic model group and drug treatment group, respectively.

NRI was calculated as average Rr score of up-, down-regulated nodes and all the nodes in the T2D network by equation (6).

6 $$ \mathrm{N}\mathrm{R}\mathrm{I}=\mathrm{mean}\left(\mathrm{Rrup},\ \mathrm{R}\mathrm{rdown},\ \mathrm{R}\mathrm{rtotal}\right) $$

In equation (6), Rr_up_, Rr_down_ and Rr_total_ refer to the Rr score of upregulated nodes, downregulated nodes and all nodes, respectively.

### Real-time quantitative RT-PCR

The microarray data was validated by real-time quantitative RT-PCR. Total RNA (1 μg) was used for cDNA synthesis, using QuantiTect Reverse Transcription Kit (Qiagen) according to the manufacturer’s instructions.

PCR reactions were carried out using QuantiFast SYBR Green PCR Kit (Qiagen) according to the manufacturer’s protocol in an Eppendorf realplex system (Hamburg, Germany). Primer sequences are listed in Additional file 1. Thermal cycling conditions were as follows: 95 °C for 5 min followed by 40 cycles at 95 °C for 10 s and 60 °C for 30 s. The relative expression level for each gene was normalized to that of β-actin and reported as mean relative changes (± SD) compared with normal controls.

### Statistical analysis

Data are expressed as mean ± SD. Significance analysis was performed using one-way analysis of variance (ANOVA) with Dunnett's post-hoc test for multiple comparison analysis, and *p* < 0.05 was regarded as statistically significant.

## Results

### Inhibition of 3T3-L1 cells differentiation by XKA

**Fig. 2:**
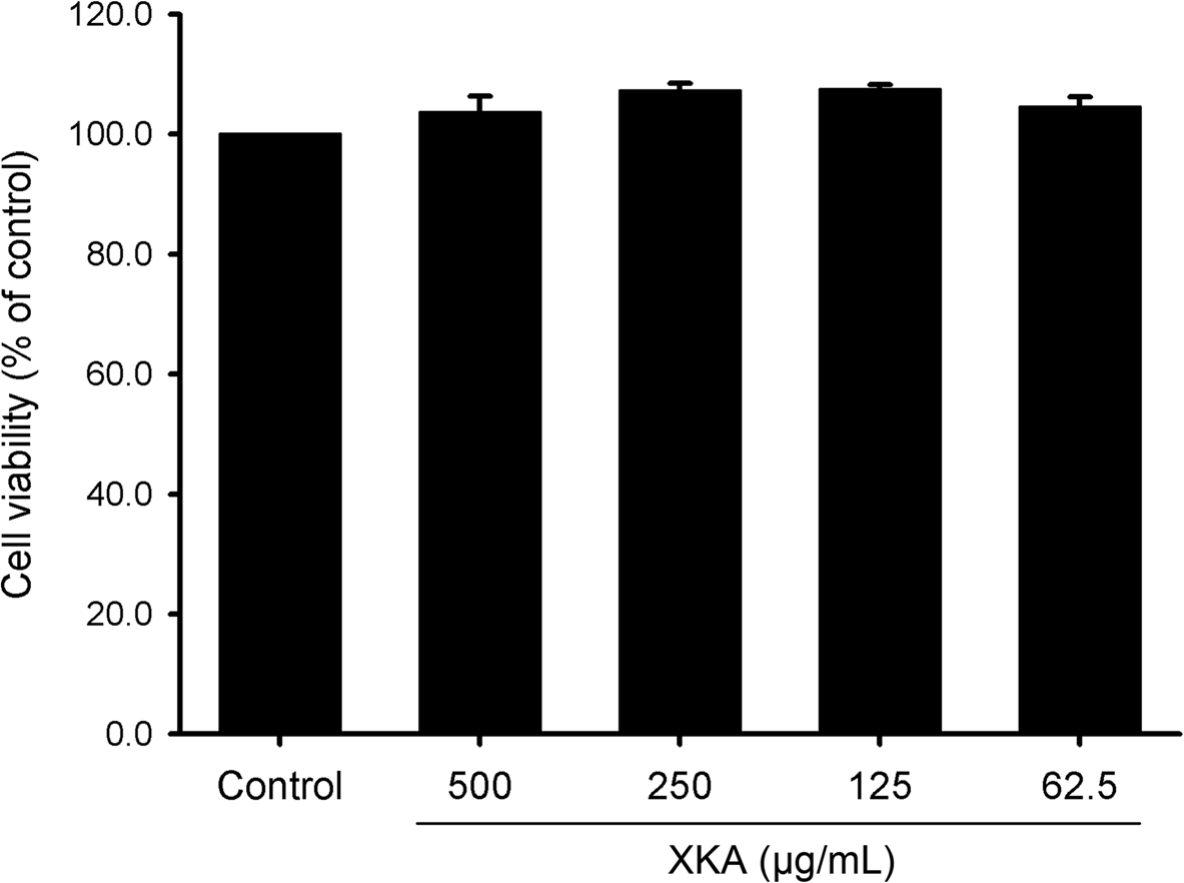
Effect of XKA on cell viability of 3T3-L1 preadipocytes. All values are represented as means ± SD (*n* = 3)

**Fig. 3:**
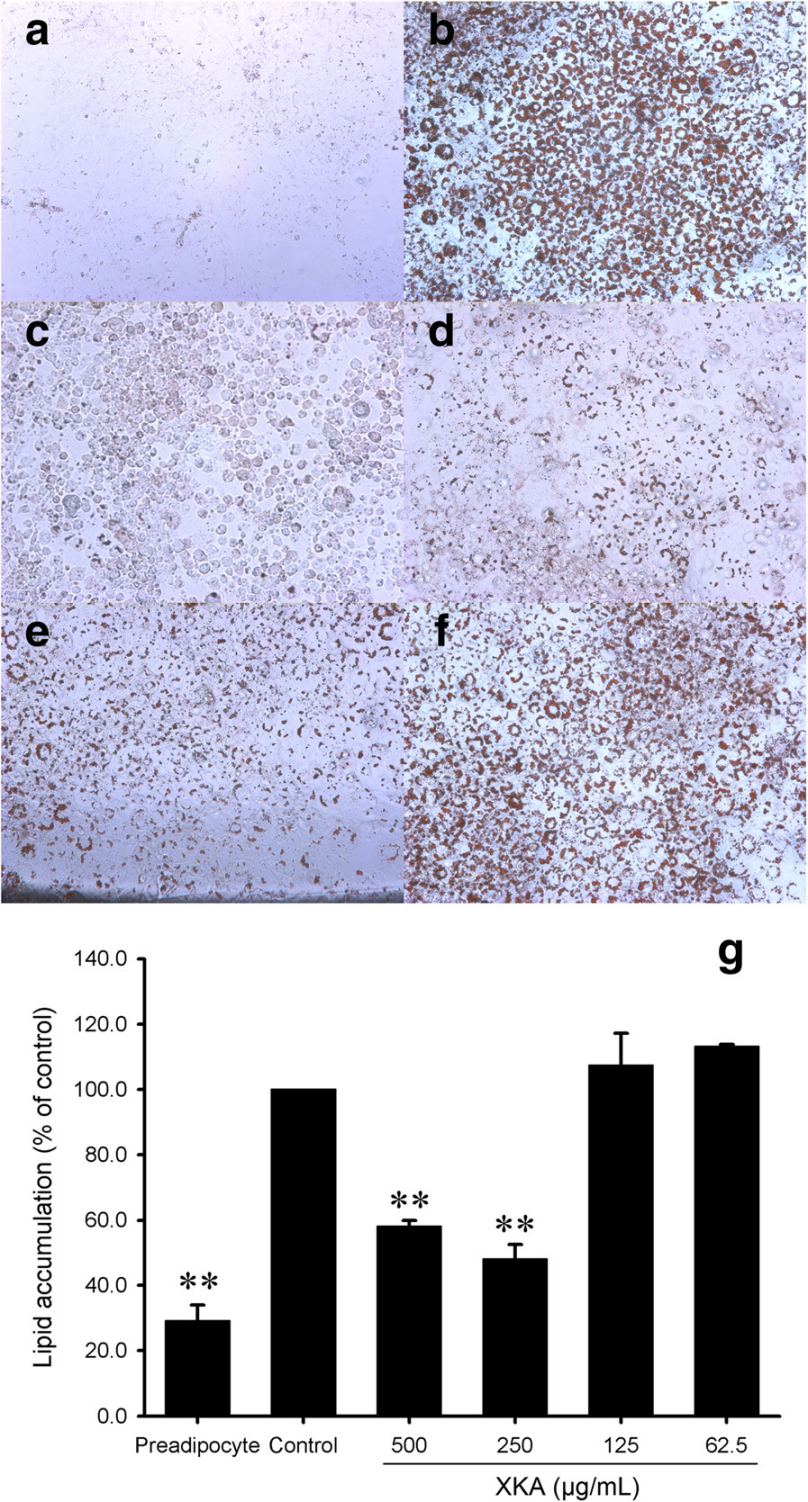
Effect of XKA on adipocyte differentiation. Representative pictures of preadipocyte (**a**) and differentiation induced cells with Oil Red O staining at XKA concentrations of 0 (**b**), 500 (**c**), 250 (**d**), 125 (**e**) and 62.5 (**f**) μg/mL (Original magnification 100 ×). Oil Red O dye in lipid droplets was eluted into isopropanol to determine the accumulation of lipid (**g**). All values are represented as means ± SD (*n* = 3). *p < 0.05 vs. control group, **p < 0.01 vs. control group

To determine the effects of XKA on 3T3-L1 cells differentiation, confluent 3T3-L1 cells were treated with different concentrations of XKA extracts. The anti-adipogenic effect of XKA was investigated at concentrations with no effect on cell viability according to MTT assay (Fig. 2). Following various treatments, Oil Red O was used to stain the differentiated 3T3-L1 cells in 24-well plates (Fig. 3). Microscopic observation of cells with Oil Red O staining indicated that XKA reduced lipid accumulation in a dose-dependent manner. This result was confirmed by quantitative data derived from spectrophotometric measure of Oil Red O-stained lipid droplets eluted by isopropanol, which demonstrated that XKA effectively inhibited adipocyte differentiation compared to control at concentrations of 500 and 250 μg/mL (*p* < 0.01, Fig. 3).

**Fig. 4:**
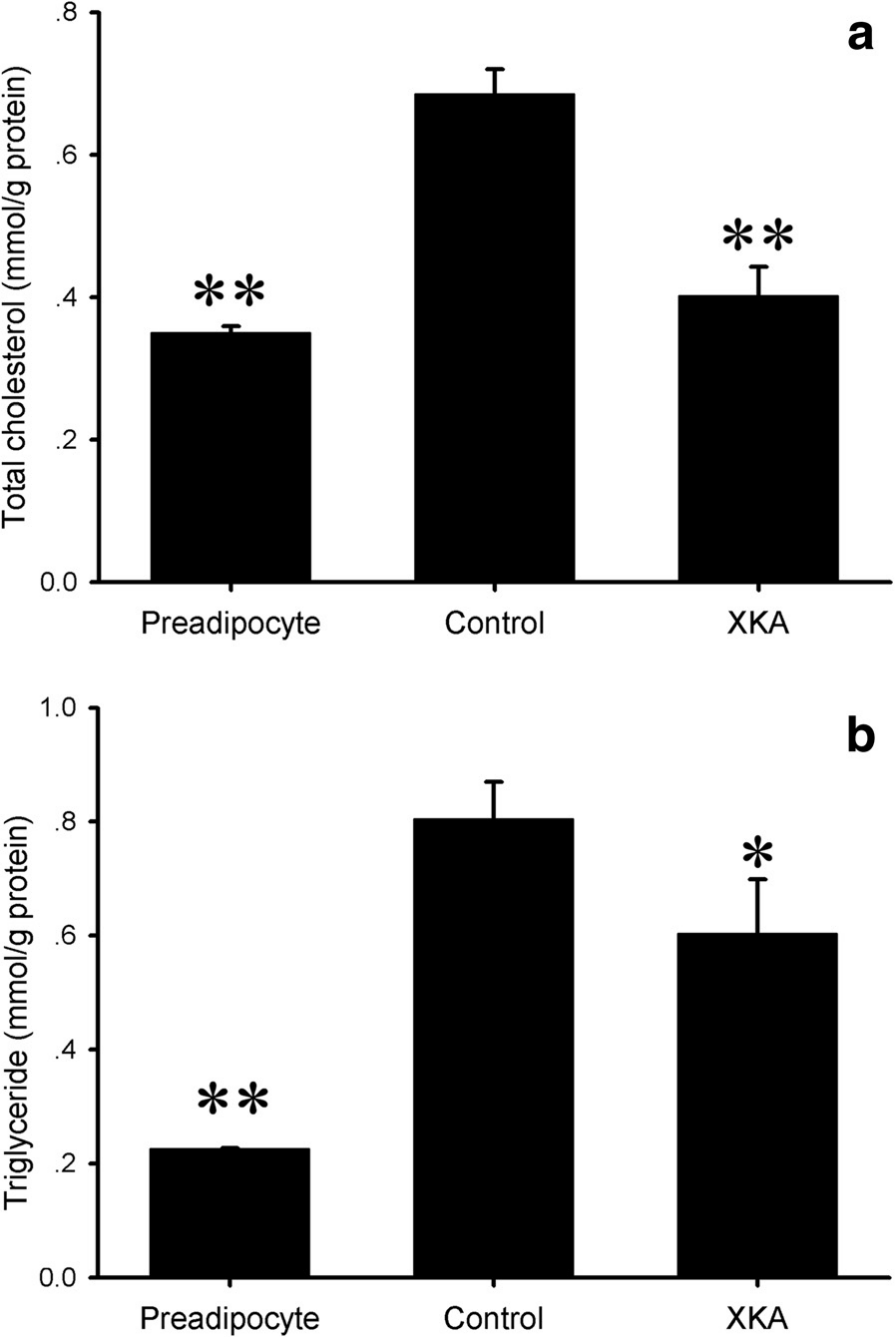
Effect of XKA on TG and TC accumulation in 3T3-L1 cells. The concentrations of XKA were both 500 μg/mL in (**a**) and (**b**). All values are means ± SD (*n* = 3). *p < 0.05 vs. control group, **p < 0.01 vs. control group

Compared to control, the intracellular TC and TG contents of XKA treated 3T3-L1 cells were lowered by 41.3 % (*p* < 0.01) and 25.0 % (*p* < 0.05), respectively (Fig. 4). Therefore, XKA treatment led to an inhibition of 3T3-L1 adipocyte differentiation.

### Effect of XKA on blood glucose levels in KKAy mice

**Fig. 5:**
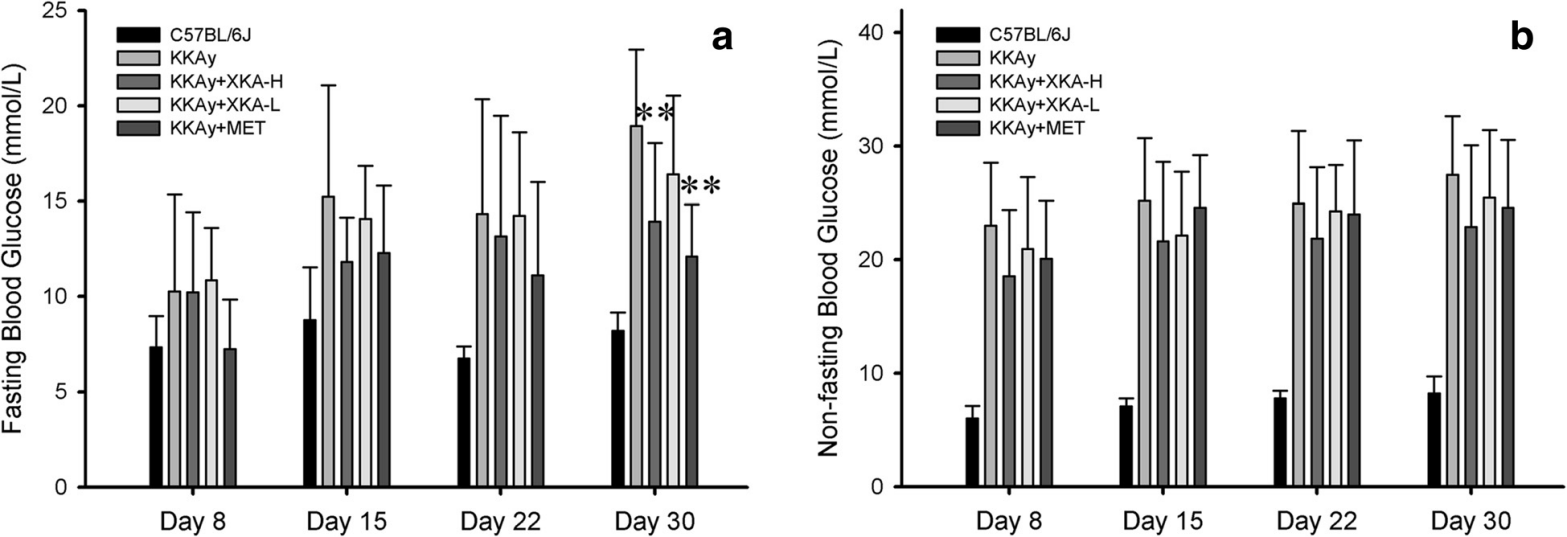
*In vivo* glucose levels down-regulation effect of XKA in KKAy mice. Fasting (**a**) and non-fasting (**b**) blood glucose was determined at indicated time. All values are means ± SD (*n* = 10–11). *p < 0.05 vs. KKAy mice, **p < 0.01 vs. KKAy mice

To evaluate the effect of XKA on blood glucose levels, the KKAy mice were treated with 0.75 g/kg XKA, 1.5 g/kg XKA, 250 mg/kg metformin or distilled water for 32 days. Blood glucose values of the diabetic model group were significantly higher than those of the C57BL/6J mice. The blood glucose attenuation effect of XKA was dose-dependent. At the 4th week treatment with 1.5 g/kg XKA, the fasting and non-fasting blood glucose levels of the KKAy mice decreased by 26.4 % (*p* < 0.01) and 16.8 %, respectively (Fig. 5). In diabetic KKAy mice, XKA at a dose of 1.5 g/kg showed similar treatment effects to that of metformin.

### Effect of XKA on oral glucose tolerance in KKAy mice

**Fig. 6:**
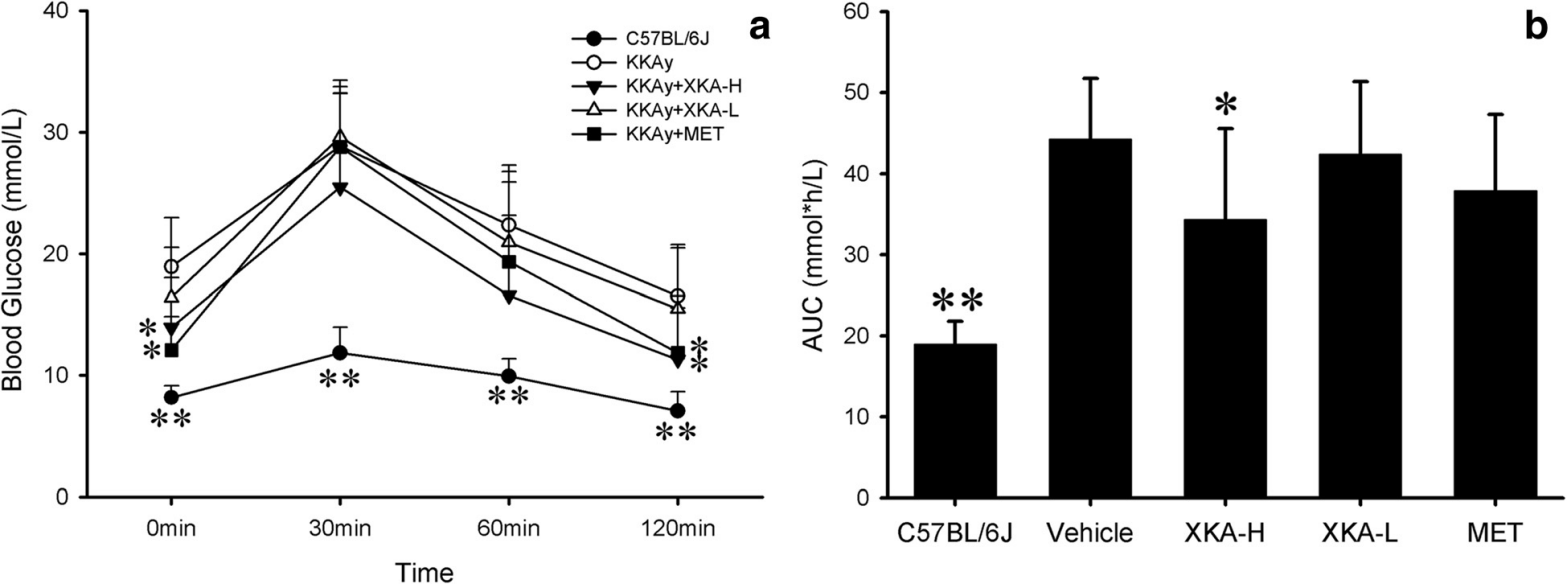
Effects of XKA on oral glucose tolerance test (OGTT). OGTT was carried out at day 30 (**a**), and the AUC of OGTT was calculated (**b**). All values are means ± SD (*n* = 10–11). *p < 0.05 vs. KKAy mice, **p < 0.01 vs. KKAy mice

In the present study, oral glucose tolerance tests were performed in the mice after 30 days of treatment to determine the whole-body insulin sensitivity. Compared with the non-diabetic controls, the diabetic model group exhibited a stronger hyperglycemic response to oral glucose administration. Significant differences in blood glucose levels over time were observed for XKA (1.5 g/kg) and metformin 120 min post glucose challenge, compared with vehicle-treated KKAy mice (Fig. 6). Moreover, the AUC in the XKA (1.5 g/kg) treated group decreased significantly compared to the vehicle-treated group. In addition, XKA improved the oral glucose tolerance in a dose-dependent manner.

### Effect of XKA on dyslipidemia in KKAy mice

To evaluate the effects of XKA on dyslipidemia, the levels of TG, TC, HDL-c and LDL-c in KKAy mice were determined, as shown in Table 1. Compared to diabetic model group, the serum lipid profile was improved to some extent by XKA treatment in KKAy mice. XKA (1.5 g/kg) and XKA (0.75 g/kg) could decrease serum TG level by 30.3 % and 18.7 %, respectively. The level of serum HDL-c was elevated by XKA (1.5 g/kg) by 24.5 %. TC-HDL-c ratio is an independent predictors of coronary heart disease (CHD) events [22]. In XKA (1.5 g/kg and 0.75 g/kg) treatment groups, TC-HDL-c ratio of KKAy mice was significantly lowered by 12.0 % and 9.2 %, respectively. The atherogenic index, as a risk factor for coronary artery disease, was significantly decreased by both doses of XKA.

**Table 1:** Effect of XKA on serum lipid profile in diabetic KKAy mice

	C57BL/6J	KKAy	KKAy + XKA-H	KKAy + XKA-L	KKAy + MET
TG (mmol/L)	1.29 ± 0.17	2.03 ± 0.85^#^	1.41 ± 0.59^*^	1.65 ± 0.33	1.64 ± 0.56
TC (mmol/L)	2.33 ± 0.21	4.61 ± 1.01^##^	4.92 ± 1.65	4.44 ± 0.83	4.85 ± 0.97
HDL-c (mmol/L)	1.42 ± 0.16	2.75 ± 0.60^##^	3.42 ± 1.42	2.94 ± 0.70	3.21 ± 0.75
LDL-c (mmol/L)	0.408 ± 0.075	0.565 ± 0.121^#^	0.617 ± 0.169	0.513 ± 0.111	0.565 ± 0.087
TC/HDL-c	1.64 ± 0.07	1.68 ± 0.13	1.48 ± 0.17^**^	1.53 ± 0.14^*^	1.53 ± 0.15^*^
Atherogenic index	0.639 ± 0.066	0.685 ± 0.127	0.483 ± 0.167^**^	0.530 ± 0.145^*^	0.533 ± 0.146^*^

All values are means ± SD (*n* = 10–11)

^#^ *p* < 0.05 vs. C57BL/6J mice

^##^ *p* < 0.01 vs. C57BL/6J mice

*p < 0.05 vs. KKAy mice

**p < 0.01 vs. KKAy mice

### Effect of XKA on body weight in KKAy mice

**Fig. 7:**
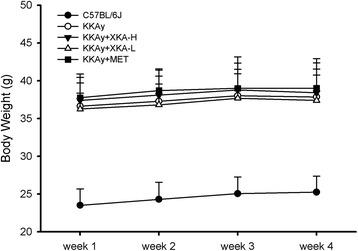
Effects of XKA on body weight in KKAy mice. All values are means ± SD (*n* = 10–11)

The effects of XKA on body weight changes were observed in KKAy mice during the treatment period. As shown in Fig. 7, the body weight of all mice increased continuously during the first three weeks, and it became stabilized in the fourth week.

The body weight gain of diabetic model group was not statistically different from that of the groups treated with XKA or metformin. The body weight of C57BL/6J was significantly lower than that of the diabetic ones.

### Effect of XKA on tissue weight in KKAy mice

The liver/body weight ratio and spleen/body weight ratio of the diabetic model was much greater than that of the lean mice. However, the relative liver and spleen weight decreased after the XKA treatment (Table 2). Especially, the relative spleen weight of the XKA-L (0.75 g/kg) treatment group showed statistical significance (*p* < 0.05), while the metformin treatment group showed a similar effect as XKA-L. The relative epidydimal fat and heart weight between the diabetic model and the treatment group didn’t exhibit statistical significant difference.

**Table 2:** Effect of XKA on tissue weight in diabetic KKAy mice

	C57BL/6J	KKAy	KKAy + XKA-H	KKAy + XKA-L	KKAy + MET
Liver (g)	1.03 ± 0.12	2.11 ± 0.35^##^	1.95 ± 0.62	1.82 ± 0.46	1.91 ± 0.30
Relative liver weight	0.042 ± 0.002	0.058 ± 0.007^##^	0.051 ± 0.011	0.049 ± 0.010	0.051 ± 0.007
Epidydimal fat pad (g)	0.23 ± 0.05	1.10 ± 0.23^##^	1.23 ± 0.22	1.29 ± 0.30	1.28 ± 0.38
Relative epidydimal fat weight	0.010 ± 0.002	0.030 ± 0.006^##^	0.033 ± 0.005	0.035 ± 0.008	0.034 ± 0.010
Perirenal fat (g)	0.05 ± 0.02	0.49 ± 0.14^##^	0.57 ± 0.19	0.58 ± 0.13	0.56 ± 0.16
Relative perirenal fat weight	0.002 ± 0.001	0.014 ± 0.004^##^	0.015 ± 0.004	0.016 ± 0.004	0.015 ± 0.004
Spleen (g)	0.07 ± 0.01	0.12 ± 0.04^##^	0.10 ± 0.02	0.10 ± 0.03	0.10 ± 0.01
Relative spleen weight	0.0027 ± 0.0004	0.0033 ± 0.0007^#^	0.0028 ± 0.0004	0.0026 ± 0.0007^*^	0.0026 ± 0.0003^*^
Kidney (g)	0.33 ± 0.04	0.47 ± 0.08^##^	0.44 ± 0.07	0.42 ± 0.04	0.42 ± 0.04
Relative kidney weight	0.014 ± 0.001	0.013 ± 0.001	0.012 ± 0.001	0.012 ± 0.001^*^	0.011 ± 0.001^**^
Heart (g)	0.13 ± 0.02	0.15 ± 0.03	0.15 ± 0.02	0.14 ± 0.01	0.15 ± 0.02
Relative heart weight	0.005 ± 0.001	0.004 ± 0.001^##^	0.004 ± 0.001	0.004 ± 0.000	0.004 ± 0.001

All values are means ± SD (*n* = 10–11)

^#^ *p* < 0.05 vs. C57BL/6J mice

^##^ *p* < 0.01 vs. C57BL/6J mice

*p < 0.05 vs. KKAy mice

**p < 0.01 vs. KKAy mice

### T2D disease network recovery effects of XKA

**Fig. 8:**
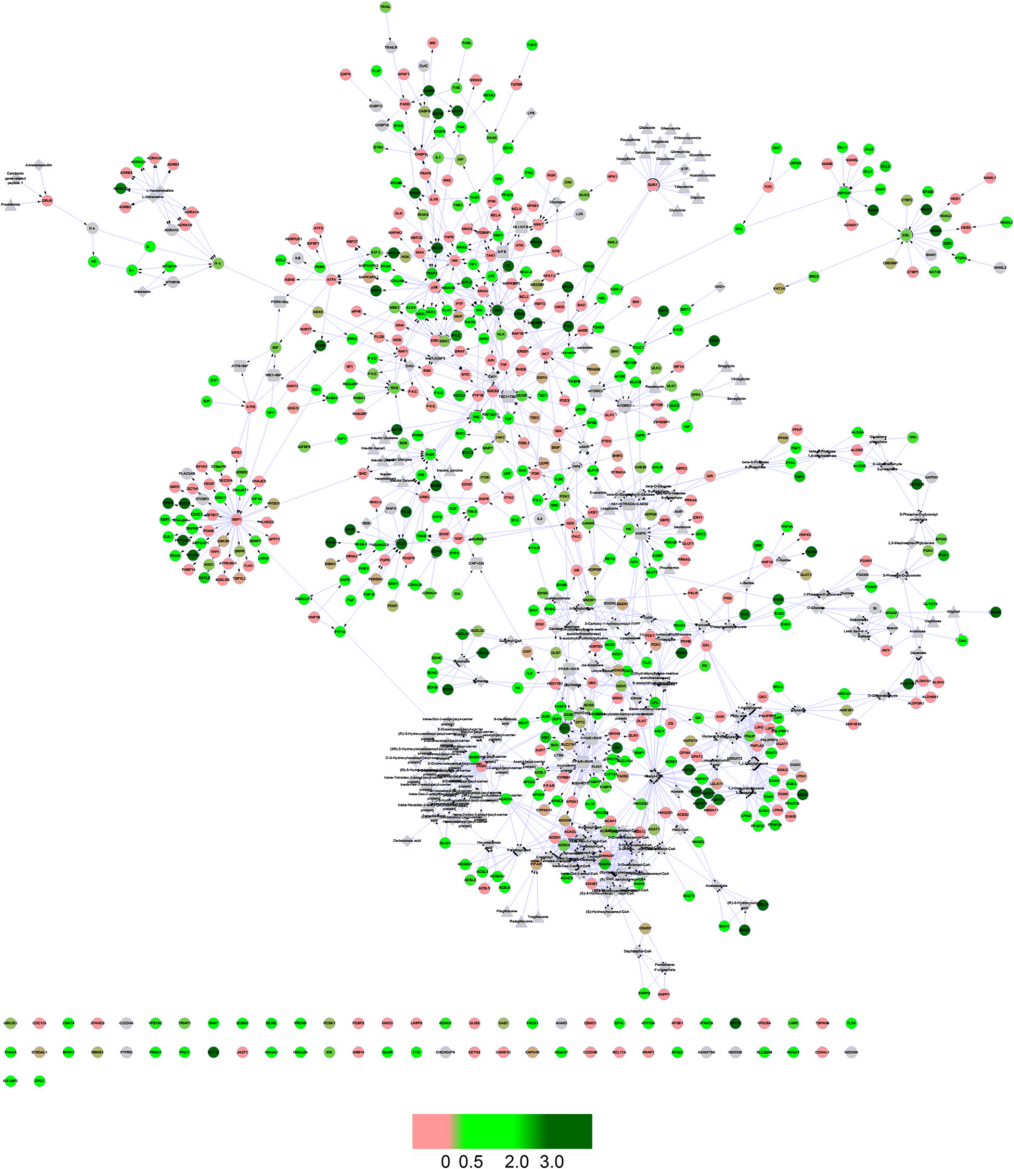
T2D network with reverse rate of XKA. This figure illustrates the reverse rate values of the nodes in the network, and the holistic reversal effect of XKA could be seen intuitively in the T2D network. The color of the nodes in the network presented the reverse rate of the nodes with the treatment of XKA. The network was visualized by Cytoscape

The RR values were mapped to the nodes in the T2D network constructed previously [21], as shown in Fig. 8. The network was visualized by Cytoscape [23]. The color of the nodes in the network presented the reverse rate of the nodes with the treatment of XKA. Therefore, the holistic reversal effect of XKA was displayed intuitively in the T2D network.

As shown in Table 3, the number of up- and down-regulated nodes by T2D was 71 and 91 in the T2D network. In total, 93.0 % (66/71) of up-regulated genes and 80.2 % (73/91) of down-regulated genes were recovered, while the 68.0 % (568/835) of all genes in the T2D network were recovered. The Rr scores of up- and down-regulated nodes showed higher degree of recovery regulation, which indicated that XKA exhibited more beneficial effects on the significantly perturbed nodes in T2D. As mentioned above, Rr_up_, Rr_down_ and Rr_total_ were 0.930, 0.802 and 0.680, respectively. Therefore, the NRI of XKA was 0.804 according to equation (6). The NRIs of the T2D-related pathways summarized in T2D@ZJU (http://tcm.zju.edu.cn/t2d) were also calculated, which were presented in Additional file 2.

**Table 3:** The regulation of the T2D network by XKA

	Number of reversed nodes	Number of nodes	Rr
Nodes up-regulated	66	71	0.930
Nodes down-regulated	73	91	0.802
Total nodes	568	835	0.680

### Verification of microarray gene expression data by real-time quantitative RT-PCR

**Fig. 9:**
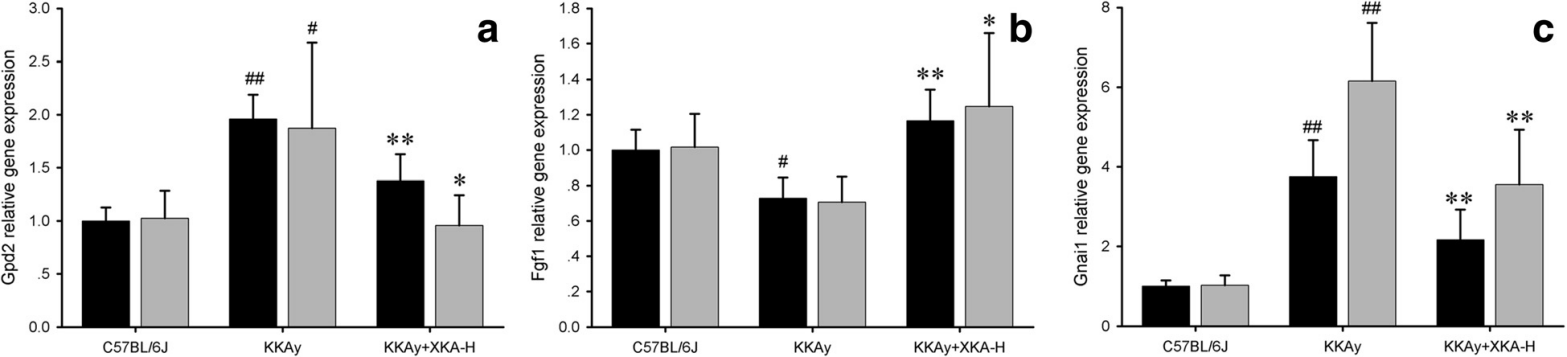
Validation of microarray gene expression data by real-time quantitative RT-PCR. Black bars indicate the relative gene expression from microarray data, while gray bars indicate that from real-time quantitative RT-PCR. (**a**) Gpd2, (**b**) Fgf1, (**c**) Gnai1. All values are means ± SD (*n* = 5). ^#^ *p* < 0.05 vs. C57BL/6J mice, ^##^ *p* < 0.01 vs. C57BL/6J mice, *p < 0.05 vs. KKAy mice, **p < 0.01 vs. KKAy mice

The expression levels of highly reversed transcripts were confirmed by real-time quantitative RT-PCR (Fig. 9). These genes include glycerol phosphate dehydrogenase 2, mitochondrial (Gpd2), fibroblast growth factor 1 (Fgf1) and guanine nucleotide binding protein (G protein), alpha inhibiting 1 (Gnai1). The real-time quantitative RT-PCR expression levels were found to be consistent among biological replicates and in similar patterns with the microarray data.

## Discussion

T2D is one of the most prevalent and serious metabolic diseases in the world characterized by disorders of glucose and lipid metabolism. TCM is receiving more attention all over the world for its outstanding efficacy as multi-target drugs with less frequent adverse effects. Unlike the common antidiabetic chemical drugs aimed at a specific single target, TCM attempted to restore the balance of a perturbed molecular disease network with integrated effects of multi-components upon multi-targets and multi-pathways. The evaluation of the antidiabetic chemical drugs is relatively simple, *e.g.* blood glucose levels should examined for anti-hyperglycemia drugs, while the blood triglyceride and total cholesterol levels should be examined to evaluate the efficacy of anti-hyperlipidemia ones. However, a single index or biomarker could barely reflect the holistic treatments and the integrated effects of TCM.

In this study, XKA was investigated for the therapeutic effects in 3T3-L1 cells and KKAy mice with both conventional methods and holistic ones. *In vitro*, XKA inhibited adipogenic differentiation, lowered the intracellular TC and TG contents in 3T3-L1 cells. In KKAy mice, XKA administration did not show any significant effect on body weight. After the treatment, average blood glucose levels of XKA treated groups were lower than vehicle control. The blood triglyceride levels of XKA treated groups were also lower than the vehicle control, while the HDL-c levels of XKA treated groups were higher. The TC-HDL-c ratio and the atherogenic index of XKA treated KKAy mice were both significantly improved. Therefore, XKA exhibited the effects of improvement in the disorders of glucose and lipid metabolism. In the OGTT, the KKAy mice given XKA (1.5 g/kg body weight) showed significant improvement in insulin sensitivity compared to vehicle control. This indicates that XKA could improve the insulin sensitivity in diabetic animal model. Taken together, treatment with XKA exhibited the effects in attenuating the disorders in glucose and lipid metabolism and alleviating the insulin resistance, both of which were important characteristics of T2D. Phenolic acids derived from *Salviae miltiorrhizae radix et rhizoma*, xanthones derived from *Anemarrhenae rhizoma*, iridoids derived from *Rehmanniae radix*, triterpenoid saponins from *Ginseng radix et rhizoma* and alkaloids from *Coptidis rhizoma* might be the principal anti-diabetic components in XKA based upon network analysis [16].

TCMs, like many other herbal medicines, exert integrated effects through multi-components. A few holistic and integrated approaches have been developed to discover the effective substances, as well as the efficacy and safety of TCMs [24, 25]. In this study, a strategy integrating network pharmacology analysis technology and ‘omics’ data was adopted. As a key tissue to regulate the metabolism related pathways, livers of these mice were collected for further systems biology research. In our previous study, a T2D associated molecular network was constructed by organizing T2D-related pathways in several well-known pathway databases. RR was used to evaluate the effects of XKA in reversing the changes of gene expression in diabetic models. The RR values were mapped to the nodes in the T2D network, as shown in Fig. 8. This result implied that XKA has a reverse effect on T2D, indicating treatment efficacy of XKA from a network perspective.

In order to quantitatively evaluate the efficacy of XKA, several index associated with network recovery were calculated. Rr_up_, Rr_down_ and Rr_total_ were 0.930, 0.802 and 0.680, respectively. The values of Rr_up_ and Rr_down_ were both higher than that of Rr_total_, which implicated that XKA exhibited greater reverse effects in the seriously deviated nodes in disease state. In this study, NRI was used to quantitatively evaluate the network rebalance effect of drug treatment in the unbalanced molecular network of T2D. The NRI was a value between 0 and 1, and a higher NRI represented a better treatment efficacy of drug in treating T2D. The NRI of XKA was calculated to be 0.804, which indicated a well-performed network rebalance effect of XKA. The NRIs of the T2D-related pathways were useful index suggesting the XKA’s probable mechanisms of action. The top 2 NRIs of these pathways derived from “Glycolysis and Gluconeogenesis” pathway and “Fatty acid biosynthesis and metabolism” pathway, with NRIs of 0.947 and 0.938, respectively. It indicated that attenuating the disorders in glucose and lipid metabolism might be XKA’s major antidiabetic mechanisms. The NRI of “Insulin Signaling” pathway was 0.897, which meant that XKA might also alleviate insulin resistance. These results were consistent with the phenotype indexes in cell and animal as mentioned above.

## Conclusion

In conclusion, XKA, as an example of TCM in treating T2D, was investigated for its therapeutic effects in 3T3-L1 cells and KKAy mice with both conventional and holistic omics methods. In 3T3-L1 cells, XKA inhibited adipogenic differentiation, lowered the intracellular TC and TG contents. In KKAy mice, XKA reduced blood glucose, serum triglyceride, increased serum HDL-c, lowered the atherogenic index and improved insulin resistance. Furthermore, the microarray data of liver was integrated with the molecular T2D network to evaluate the efficacy of XKA at a systems level. The results indicated an effective therapy of XKA by reversing the molecular T2D disease network from a perturbed state. Integrating the conventional and the holistic omics approach, the antidiabetic effect of XKA was evaluated more comprehensively to provide both phenotypic evidence and underlying action mechanisms for the clinical use of XKA treating T2D.

## Additional files

Additional file 1: Table S1. List of primers used in real-time quantitative RT-PCR analysis. (DOCX 13 kb) Additional file 2:
**The regulation of the T2D-related pathways by XKA.** (DOCX 13 kb)

## Abbreviations

XKA: Xiao-Ke-An
T2D: Type 2 diabetes mellitus
TC: Total cholesterol
TG: Triglyceride
HDL-c: High-density lipoprotein cholesterol
LDL-c: Low-density lipoprotein cholesterol
TCM: Traditional Chinese medicines
BMI: Body mass index
HbA1c: Glycated haemoglobin
DMSO: Dimethyl sulfoxide
MTT: 3-(4,5-dimethylthiazol-2-yl)-2,5-diphenyltetrazolium bromide
DMEM: Dulbecco's Modified Eagle Medium
FBS: Fetal bovine serum
PFA: Paraformaldehyde
NRI: Network recovery index
RS: Regulating score
ANOVA: Analysis of variance
RR: Reverse rate
ASNS: Asparagine synthetase
