# Supplementary material for: Evaluating the antidiabetic effects of Chinese herbal medicine: Xiao-Ke-An in 3T3-L1 cells and KKAy mice using both conventional and holistic omics approaches
Source: BMC Complement Altern Med. 2015 Aug 13;15:272. doi: 10.1186/s12906-015-0785-2 (PMC4534019; doi:10.1186/s12906-015-0785-2)
Supplement: Additional file 1: Table S1. — List of primers used in real-time quantitative RT-PCR analysis. (DOCX 13 kb) [file 12906_2015_785_MOESM1_ESM.docx]

| Table S1. List of primers used in real-time quantitative RT-PCR analysis | | |
| --- | --- | --- |
| Gene | Forward Primer | Reverse Primer |
| Gpd2 | GAAGGGGACTATTCTTGTGGGT | GGATGTCAAATTCGGGTGTGT |
| Fgf1 | CCCTGACCGAGAGGTTCAAC | GTCCCTTGTCCCATCCACG |
| Gnai1 | GGTTTACAGACACGTCCATCAT | GCCTGCATATTCTGGGTAGCAT |
