# Supplementary material for: Evaluating the antidiabetic effects of Chinese herbal medicine: Xiao-Ke-An in 3T3-L1 cells and KKAy mice using both conventional and holistic omics approaches
Source: BMC Complement Altern Med. 2015 Aug 13;15:272. doi: 10.1186/s12906-015-0785-2 (PMC4534019; doi:10.1186/s12906-015-0785-2)
Supplement: Additional file 2: — The regulation of the T2D-related pathways by XKA. (DOCX 13 kb) [file 12906_2015_785_MOESM2_ESM.docx]

**Additional file 1. The regulation of the T2D-related pathways by XKA.**

| Pathway | NRI |
| --- | --- |
| AMPK Signaling | 0.794 |
| ERK Signaling | 0.811 |
| IGF-1 Signaling | 0.909 |
| Incretin Signaling | 0.879 |
| Insulin Signaling | 0.897 |
| JNK Signaling | 0.794 |
| mTOR Signaling | 0.774 |
| p38 Signaling | 0.833 |
| Unfolded Protein Response | 0.596 |
| WNT Signaling | 0.781 |
| Citrate cycle | 0.813 |
| Fatty acid biosynthesis and metabolism | 0.938 |
| Glycerolipid metabolism | 0.760 |
| Glycolysis and Gluconeogenesis | 0.947 |
| Notch signaling | 0.928 |
